# Type of vaccine and immunosuppressive therapy but not diagnosis critically influence antibody response after COVID-19 vaccination in patients with rheumatic disease

**DOI:** 10.1136/rmdopen-2022-002650

**Published:** 2022-12-06

**Authors:** Leonie Maria Frommert, Amanthi Nadira Arumahandi de Silva, Jan Zernicke, Veronika Scholz, Tanja Braun, Lara Maria Jeworowski, Tatjana Schwarz, Pinkus Tober-Lau, Alexander ten Hagen, Elisa Habermann, Florian Kurth, Leif Erik Sander, Victor Max Corman, Gerd-Rüdiger Burmester, Robert Biesen, Fredrik N. Albach, Jens Klotsche

**Affiliations:** 1 Department of Rheumatology and Clinical Immunology, Charité – Universitätsmedizin Berlin, corporate member of Freie Universität Berlin and Humboldt Universität zu Berlin, Berlin, Germany; 2 Institute of Virology, Charité – Universitätsmedizin Berlin, corporate member of Freie Universität Berlin and Humboldt Universität zu Berlin, Berlin, Germany; 3 German Centre for Infection Research (DZIF), Associated Partner Site, Berlin, Germany; 4 Department of Infectious Diseases and Respiratory Medicine, Charité – Universitätsmedizin Berlin, corporate member of Freie Universität Berlin and Humboldt Universität zu Berlin, Berlin, Germany; 5 Labor Berlin, Charité - Vivantes GmbH, Berlin, Germany; 6 Epidemiology Unit, German Rheumatism Research Center Berlin – a Leibniz Institute (DRFZ), Berlin, Germany

**Keywords:** rituximab, COVID-19, vaccination, autoimmune diseases, arthritis, rheumatoid

## Abstract

**Objective:**

The development of sufficient COVID-19 vaccines has been a big breakthrough in fighting the global SARS-CoV-2 pandemic. However, vaccination effectiveness can be reduced in patients with autoimmune rheumatic diseases (AIRD). The aim of this study was to identify factors that lead to a diminished humoral vaccination response in patients with AIRD.

**Methods:**

Vaccination response was measured with a surrogate virus neutralisation test and by testing for antibodies directed against the receptor-binding-domain (RBD) of SARS-CoV-2 in 308 fully vaccinated patients with AIRD. In addition, 296 immunocompetent participants were investigated as a control group. Statistical adjusted analysis included covariates with a possible influence on antibody response.

**Results:**

Patients with AIRD showed lower antibody responses compared with immunocompetent individuals (median neutralising capacity 90.8% vs 96.5%, p<0.001; median anti-RBD-IgG 5.6 S/CO vs 6.7 S/CO, p<0.001). Lower antibody response was significantly influenced by type of immunosuppressive therapy, but not by rheumatic diagnosis, with patients under rituximab therapy developing the lowest antibody levels. Patients receiving mycophenolate, methotrexate or janus kinase inhibitors also showed reduced vaccination responses. Additional negative influencing factors were vaccination with AZD1222, old age and shorter intervals between the first two vaccinations.

**Conclusion:**

Certain immunosuppressive therapies are associated with lower antibody responses after vaccination. Additional factors such as vaccine type, age and vaccination interval should be taken into account. We recommend antibody testing in at-risk patients with AIRD and emphasise the importance of booster vaccinations in these patients.

**WHAT IS ALREADY KNOWN ON THIS TOPIC**

- COVID-19 vaccination effectiveness can be reduced in patients with autoimmune rheumatic diseases (AIRD).
- There is uncertainty regarding the independent impact of various influencing factors on vaccination response in patients with AIRD and which patients would benefit from antibody testing.

**WHAT THIS STUDY ADDS**

- A lower antibody response after two COVID-19 vaccinations among patients with AIRD can be attributed to immunosuppressive therapies such as rituximab, mycophenolate, methotrexate or janus kinase inhibitors, but not to the rheumatic diagnosis itself.
- Old age (≥60 years), vaccination with two doses of AZD1222 and a short time period between administration of first and second vaccination are further negative influencing factors for antibody response.

**HOW THIS STUDY MIGHT AFFECT RESEARCH, PRACTICE OR POLICY**

- Antibody testing and additional booster vaccinations should be considered for patients with AIRD who are at risk of decreased antibody responses.

## Introduction

Patients with autoimmune rheumatic diseases (AIRD) are at a slightly higher risk for infection with SARS-CoV-2 and for a more severe outcome of COVID-19 compared with healthy individuals.^1^ Thus, the development of effective COVID-19 vaccines has been an important breakthrough. However, it is also known that vaccination effectiveness can be reduced in patients with AIRD,^2 3^ raising the need for a strategy to identify patients who might benefit from antibody testing and additional vaccine doses.

Since patients with AIRD have been largely excluded from the vaccination registration studies, data needed to be collected to fill the knowledge gap regarding COVID-19 vaccination in rheumatic patients. Immunogenicity of different vaccines has since been studied in a number of AIRD cohorts, but common limitations of previous research include small cohorts,^4 5^ non-consideration of covariates,^6 7^ lack of a control group^7–9^ or restriction to a singular disease^10^ or therapy.^11 12^ Also, analyses have often focused on vaccination responder rates,^7 13^ which are based on cut-offs defined by the test manufacturers after validation for detection of previous infection, but not for evaluation of vaccination response. It has since become evident that low values within the responder range can impact immunoprotection as well.^14 15^

While certain rheumatic therapies such as rituximab (RTX), mycophenolate (MMF) and methotrexate (MTX) have been shown to reduce immunogenicity in patients after vaccination,^7 13 16 17^ unanswered questions remain regarding the role of more rarely used therapies or those with a more subtle influence, as well as the potential influence of the rheumatic diagnosis itself or of additional risk factors in patients with AIRD.

Knowing the factors that influence vaccination response is important for identifying patients with AIRD at risk of insufficient humoral protection against SARS-CoV-2. We therefore aimed to identify the factors that lead to a diminished humoral response and investigated the immunogenicity of different COVID-19 vaccines in a large cohort of patients with AIRD, using an immunocompetent control group (IC) for comparison.

## Methods

### Study design and recruitment of participants

This report depicts results of the VACCIMMUN Study,^16^ which is a retrospective cohort study among patients with AIRD at Charité Medical Clinic for Rheumatology and Clinical Immunology. Clinical characterisation and blood sampling took place between June and September 2021. In addition, stored blood samples collected in April and May 2021 were included. Information regarding medical history, including comorbidities, COVID-19 vaccination status and immunosuppressive therapy, was provided directly by patients and additionally validated with medical records. Participants had to meet the following inclusion criteria: age 18 years or older, AIRD diagnosis and vaccination with a COVID-19 vaccine authorised for use in Germany. Patients with prior SARS-CoV-2 infection, identified by nucleocapsid antibody testing and patient interviews, were excluded from this investigation. Pausing of medication around the vaccinations was recorded, but the study did not propose any pausing schemes.

For data analysis, patients were assigned to mutually exclusive established broad categories as well as specific subgroups of rheumatic diagnoses and immunosuppressive therapies. In case of overlap syndromes, only the primary diagnosis was considered. Monotherapy groups were formed, and for combination therapy groups (eg, MTX+others, leflunomide+others) only drugs with no or a significantly lesser expected impact on vaccination response (eg, combination with anti-TNFα antibodies or hydroxychloroquine)^18^ were allowed as combination treatments (online supplemental table 1). Remaining individual therapy regimens with less than three patients each were grouped and categorised as ‘others’. Combination therapy with a maximum of 5 mg prednisolone/day was allowed. In addition to patients with AIRD, participants from three other cohort studies (EICOV, COVIMMUNIZE and COVIM) conducted at Charité - Universitätsmedizin Berlin served as immunocompetent controls (IC; healthcare workers and elderly patients without AIRD diagnosis or immunosuppressive medication) 19,20.

10.1136/rmdopen-2022-002650.supp1 Supplementary data

### Laboratory analyses

Antibody response was measured predominantly about 2–4 weeks after the second dose of vaccination. Maximum time from vaccination to blood taking was restricted to 60 days to avoid an influence of waning antibody responses. Neutralising antibody levels were assessed using a surrogate virus neutralisation test (cPass Neutralisation, GenScript, distributed by Medac GmbH, Wedel, Germany)^21^ with a manufacturer-defined cut-off for positivity at ≥30%. In addition, IgG antibodies against nucleocapsid, receptor binding domain (RBD), full spike and the S1 domain of the spike protein were tested using SeraSpot Anti-SARS-CoV-2 IgG microarray-based immunoassay (Seramun Diagnostica, Heidesee, Germany) and served as further validation. The threshold for positivity for anti-SARS-CoV-2 IgG levels was set at >1.00 S/CO (signal/predefined cut-off of 30) in accordance with manufacturer’s instructions. All analyses were performed using neutralisation capacities as well as anti-RBD-IgG levels.

### Statistical analysis

Descriptive statistics included median with IQR and absolute and relative frequencies. Spearman’s rank correlation was used to test for univariate correlations.

For the statistical evaluation of humoral vaccination success, we employed a two-part model commonly used in health economics^22^, since the differences between test results are not meaningful in coalescing high range values. First, a high humoral vaccination response was defined by a cut-off of 90% for neutralising capacity and 4.6 S/CO for anti-RBD-IgG (which corresponds to 506 BAU/mL according to the test manufacturer’s correlation measurements and thereby to approximately 80% vaccine efficacy against wild type SARS-CoV-2^14^). Second, the likelihood for vaccination success was analysed by means of a logistic regression model together with a linear regression analysis for the analyses of continuously distributed test results below the cut-offs. A Wald test was performed after fitting the two-part model in order to test whether a parameter was jointly significant in both parts of the model. The results of this combined test were reported.

We performed unadjusted (univariate) and adjusted (multivariable) analyses within patients with AIRD and patients with AIRD including IC. Results for influence of demographics, comorbidities and vaccine types on humoral vaccination success are given within patients with AIRD, whereas influence of diagnosis and therapy are analysed in patients with AIRD compared with IC. This analysis was performed once with broad disease and therapy categories and once with specific subgroups. Multivariable analysis included the covariates age, sex, body mass index (BMI), type of vaccination, vaccine interval in days, interval between second vaccination and antibody testing in days, rheumatic diagnosis, comorbidity (cardiovascular disease, type 2 diabetes, respiratory disease), immunosuppressive therapy, additional intake of prednisolone and pausing of any immunosuppressive therapy. Direct comparison with IC did not include additional prednisolone intake and pausing of immunosuppression as pausing of immunosuppression was not applicable and use of prednisolone was excluded in IC.

Patients with AIRD and IC showed differences in confounders such as age, vaccination interval and time to blood taking. Preanalyses were performed to visually test whether the distributions of confounders in patients with AIRD and IC showed a sufficient overlap in order to provide valid estimates in the adjusted comparison of patients with AIRD and IC. This analysis was conducted by plotting the probability density functions using univariate kernel density estimation.^23^

An additional subanalysis further investigated the impact of the rheumatic diagnosis on vaccination responses within the largest therapy subgroups (MTX treated patients and anti-TNFα monotherapy patients).

To specifically analyse the impact of the vaccine interval between first and second vaccination, only patients vaccinated with BNT162b2 were considered because of differing vaccination timeline recommendations between vaccines.

Statistical analyses were performed using GraphPad Prism V.9.4.0 and STATA V.12.1.

## Results

### Patient characteristics

Of 394 patients with AIRD who were initially recruited, 19 patients were excluded for different reasons (see online supplemental figure 1). Another 67 patients were excluded due to unacceptably short or long intervals from vaccination to blood collection (<12 days or >60 days after second vaccination). Hence, 308 patients with AIRD were included in this analysis (median age 59, 67.9% female). None of the patients received prophylactic antibodies against SARS-CoV-2 during the observed vaccination period. Detailed clinical characterisation is given in table 1. In addition, a cohort consisting of 296 immunocompetent healthcare workers and elderly patients (median age 40, 67.2% female) served as a control group (IC, table 1).

**Table 1:** Characteristics of patients with AIRD and immunocompetent controls

Variable	AIRD all (n=308)	IC (n=296)
Demographics		
Age, median (IQR)	59.0 (46.3–66.0)	40.0 (31.0–60.0)
Age ≥60 years, n (%)	147 (47.7)	76 (25.7)
Female sex, n (%)	209 (67.9)	199 (67.2)
BMI, median (IQR)*	25.0 (22.2–28.78)	–
Comorbidities		
Cardiovascular disease, n (%)	118 (38.3)	69 (23.3)
Type 2 diabetes, n (%)	23 (7.5)	13 (4.4)
Respiratory disease, n (%)	41 (13.3)	32 (10.8)
Vaccination		
BNT162b2, n (%)	233 (75.6)	173 (58.4)
mRNA-1273, n (%)	27 (8.8)	0
AZD1222, n (%)	22 (7.1)	32 (10.8)
AZD1222+mRNA, n (%)	26 (8.4)	91 (30.7)
AZD1222+BNT162b2	20	91
AZD1222+mRNA-1273	6	0
Vaccine interval in days, median (IQR)	41.0 (28.3–42.0)	21.0 (21.0–71.0)
Second vaccination to testing in days, median (IQR)	18.0 (14.0–33.8)	26.0 (21.0–28.0)
Vaccination response		
Neutralising capacity (%), median (IQR)	90.8 (54.2–95.9)	96.5 (93.5–97.1)
Anti-RBD-IgG (S/CO), median (IQR)	5.6 (2.0–6.6)	6.7 (6.3–7.1)
Positive neutralising capacity, n (%)†	258 (83.8)	292 (98.6)
Positive anti-RBD-IgG level, n (%)‡	246 (79.9)	293 (99.0)

*Data were only available for patients with AIRD.

†Defined as neutralising capacity against SARS-CoV-2 ≥30%.

‡Defined as anti-RBD-IgG levels >1.0 S/CO.

AIRD, autoimmune rheumatic diseases; BMI, body mass index; IC, immunocompetent controls; S/CO, signal/cut-off.

### Reduced vaccination response in patients with AIRD

**Figure 1:**
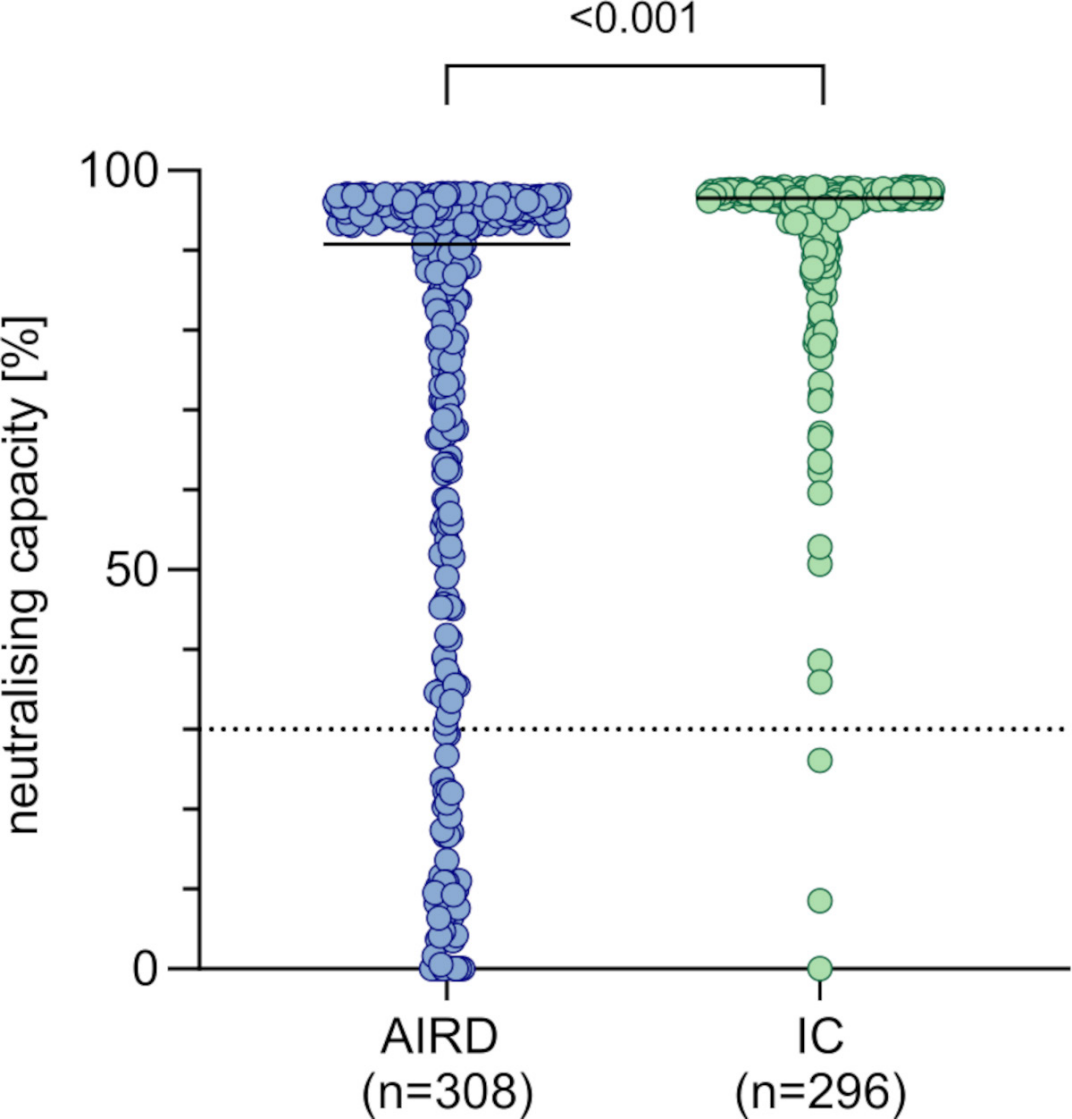
Comparison of neutralising capacity after second COVID-19 vaccination in patients with autoimmune rheumatic diseases (AIRD) and immunocompetent controls (IC). Dotted line marks the cut-off value for positivity following manufacturer’s protocol (≥30%). P values were estimated by a Wald test as combined p value of an unadjusted two-part model.

Patients with AIRD showed a significantly lower neutralising capacity after second vaccination (median 90.8%) than the IC group (median 96.5%, p<0.001, figure 1). This was also the case for anti-RBD-IgG levels (median AIRD 5.6 S/CO, median IC 6.7 S/CO, p<0.001, online supplemental figure 2). Only 1.4% of ICs showed a negative vaccination response (neutralising capacity <30%) compared with 16.2% of patients with AIRD (p<0.001, table 1). The significant difference between antibody responses of AIRD and IC remained true in adjusted analysis including the covariates age, sex, BMI, type of vaccination, vaccine interval, interval between second vaccination and antibody testing and comorbidity (p<0.001).

### Influence of type of vaccine and vaccination intervals on antibody response in patients with AIRD

**Figure 2:**
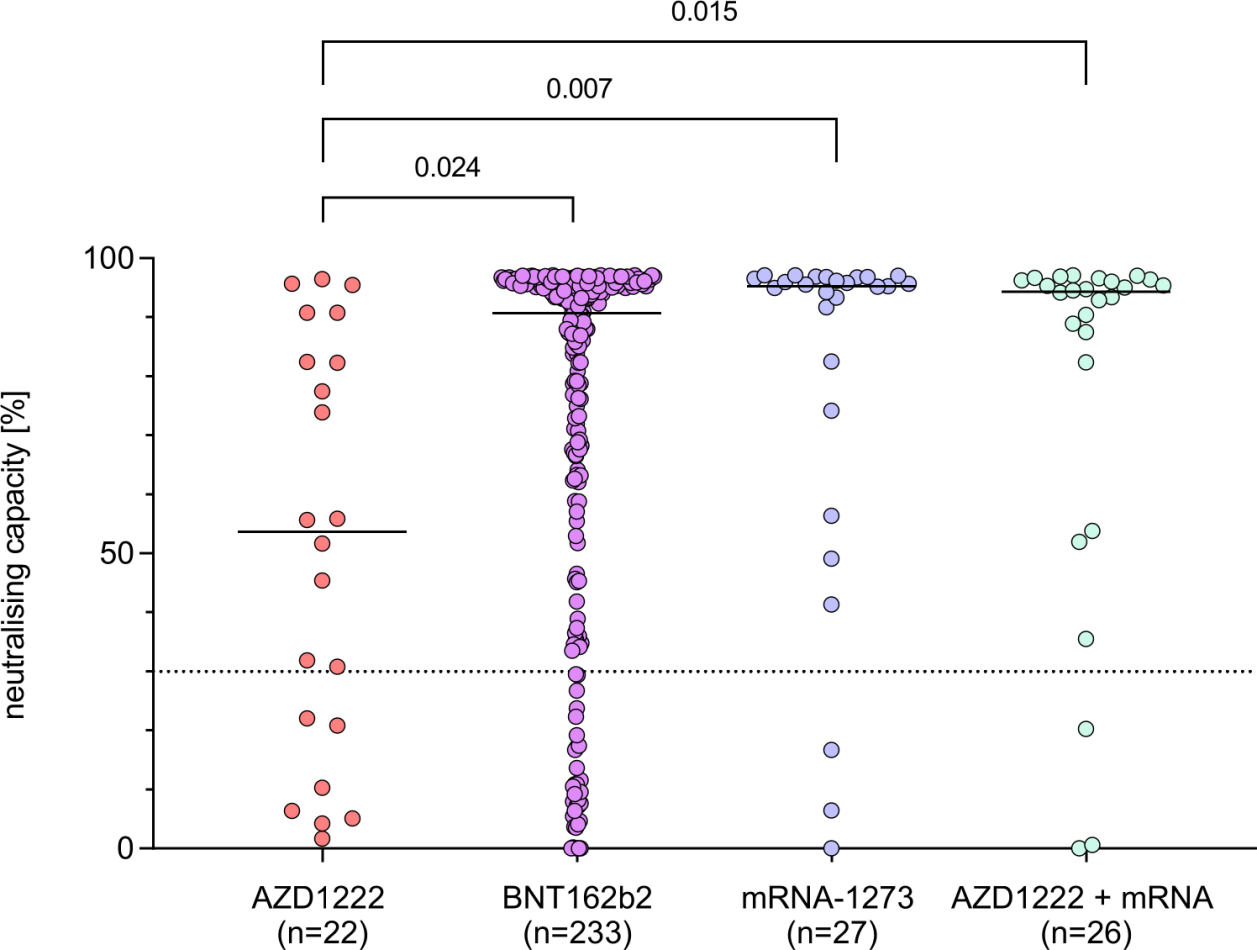
Neutralising capacity after second COVID-19 vaccination in patients with AIRD, differentiated by vaccination regime. Dotted line marks the cut-off value for positivity following manufacturer’s protocol (≥30%). P values were estimated by a Wald test as combined p value of an unadjusted two-part model. AIRD, autoimmune rheumatic diseases.

Of the patients with AIRD, 84.4% had been vaccinated with two doses of an mRNA vaccine (BNT162b2, n=233; mRNA-1273, n=27), while 7.1% of patients had received two doses of AZD1222 (n=22) and 8.4% of patients one dose of AZD1222 followed by one dose of an mRNA vaccine (n=26; table 1). Patients with AIRD vaccinated with two doses of AZD1222 showed significantly lower neutralising capacity and anti-RBD-IgG levels (53.7%, 2.0 S/CO) than those vaccinated with mRNA based vaccines (BNT162b2: 90.7%, 5.5 S/CO; mRNA-1273: 95.3%, 6.0 S/CO) or a heterologous vaccination scheme (94.4%, 6.0 S/CO, figure 2, online supplemental figure 3, table 2). Despite slightly lower median results, BNT162b2 was not associated with significantly lower neutralisation capacity or antibody response than mRNA-1273 or heterologous vaccinations in our cohort.

**Table 2:** The impact of demographics, comorbidities and vaccine type on neutralising capacity and anti-RBD-IgG levels in patients with AIRD (n=308)

Variable	Neutralising capacity (%)			Anti-RBD-IgG (S/CO)		
	Median (IQR)	Unadjusted p value	Adjusted p value	Median (IQR)	Unadjusted p value	Adjusted p value
Demographics						
Age	–	**<0.001**	0.058	–	**<0.001**	**0.001**
Age ≥60 years, n=147	82.4 (41.3– 95.3)	**0.001**	–	4.8 (0.8– 6.2)	**0.006**	–
Female sex, n=209	92.7 (62.5– 96.1)	0.052	0.106	5.8 (2.4– 6.7)	0.092	0.151
BMI	–	0.491	0.954	–	0.530	0.558
Comorbidities						
Cardiovascular disease, n=118	87.9 (38.5– 95.2)	0.176	0.518	5.3 (0.7– 6.4)	0.321	0.841
Type 2 diabetes, n=23	82.3 (16.7– 94.7)	0.425	0.691	4.4 (0.1– 6.2)	0.435	0.834
Respiratory disease, n=41	87.8 (34.5– 95.5)	0.422	0.385	5.4 (0.7– 6.4)	0.355	0.407
Vaccination						
AZD1222, n=22	53.7 (18.2– 84.5)	(ref)	(ref)	2.0 (0.3– 4.9)	(ref)	(ref)
BNT162b2, n=233	90.7 (58.9– 95.9)	**0.024**	**0.029**	5.5 (2.3– 6.5)	**0.006**	**<0.001**
mRNA-1273, n=27	95.3 (74.2– 96.8)	**0.007**	**0.043**	6.0 (3.7– 6.9)	**0.003**	**0.001**
AZD1222+mRNA, n=26	94.4 (75.2– 96.3)	**0.015**	**0.038**	6.0 (3.0– 6.8)	**0.004**	**0.026**
Vaccine interval in days	–	0.400	0.269	–	0.251	**0.002**
BNT162b2: vaccine interval >28 days, n=165	93.4 (69.1– 96.3)	0.265	–	5.9 (3.6– 6.8)	**0.048**	–
Second vaccination to testing in days	–	**0.032**	0.289	–	**0.021**	0.357

P values were estimated by a Wald test as combined p value of the two-part model. Statistically significant results in bold.

Adjusted multivariable analysis includes the covariates age, sex, BMI, type of vaccination, vaccine interval in days, interval between second vaccination and antibody testing in days, rheumatic diagnosis, comorbidity, immunosuppressive therapy, additional intake of prednisolone and pausing of any immunosuppressive therapy.

AIRD, autoimmune rheumatic diseases; BMI, body mass index; RBD, receptor-binding domain; ref, reference; S/CO, signal/cut-off.

Among patients vaccinated with BNT162b2 (n=233, vaccine interval range 21–54 days), a longer vaccine interval was associated with a higher neutralising capacity (Spearman’s rank correlation, r=0.20, p=0.002) and higher anti-RBD-IgG levels (Spearman’s rank correlation, r=0.29, p<0.001) in unadjusted correlation. When dividing these patients into two groups defined by vaccine interval ≤28 days or >28 days, a statistically significant difference between these groups was observed for anti-RBD-IgG levels (median 3.9 S/CO vs 5.9 S/CO, p=0.048, online supplemental figure 4B), but not for neutralising capacity (median 77.5% vs 93.4%, p=0.265, online supplemental figure 4A) in unadjusted analysis. The same was true for the adjusted analysis with vaccine interval as a continued variable (neutralising capacity p=0.269, anti-RBD-IgG levels p=0.002).

Time from vaccination to blood sampling (range: patients with AIRD 12–50 days, IC 18–51 days) was not significantly associated with the level of antibodies in adjusted analysis within our cohort (table 2).

### Influence of demographics and comorbidities on antibody response in patients with AIRD

Older age was associated with a lower antibody response (Spearman’s rank correlation, r=−0.34, p<0.001 for neutralising capacity and r=−0.32, p<0.001 for anti-RBD-IgG) in unadjusted correlation. When comparing patients younger than 60 years and ≥60 years, a significant difference in neutralising capacity (median 94.2% vs 82.4%, p=0.001, online supplemental figure 5A) and anti-RBD-IgG levels (median 6.0 S/CO vs 4.8 S/CO, p=0.006, online supplemental figure 5B) was observed in unadjusted analysis. Age as continuous variable remained significantly associated with lower antibody response in adjusted analysis for anti-RBD-IgG levels (p=0.001), but not for neutralising capacity (p=0.058, table 2).

There was no significant effect of sex, BMI or relevant comorbidities on antibody levels in our analysis (table 2).

### Rheumatic diagnosis did not influence antibody response

The most common rheumatic diagnoses in the AIRD cohort were rheumatoid arthritis (n=128, 41.6%), psoriatic arthritis (n=45, 14.6%) and systemic lupus erythematosus (n=33, 10.7%). A detailed list of all diagnoses can be found in table 3.

**Table 3:** The impact of rheumatic diagnosis on neutralising capacity and anti-RBD-IgG levels in patients with AIRD (n=308) compared with controls (n=296)

**Rheumatic diagnosis, n**	**Neutralising capacity (%**)			**Anti-RBD-IgG (S/CO**)		
	**Median (IQR**)	**Unadjusted** **p value**	**Adjusted** **p value**	**Median (IQR**)	**Unadjusted** **p value**	**Adjusted** **p value**
Immunocompetent controls, n=296	96.5 (93.5–97.1)	(ref)	(ref)	6.7 (6.3–7.1)	(ref)	(ref)
Rheumatoid arthritis, n=128	76.9 (35.6–92.9)	**<0.001**	0.888	5.1 (0.9–6.4)	**<0.001**	0.666
Psoriatic arthritis, n=45	95.3 (83.6–96.7)	0.108	0.583	5.9 (3.6–6.9)	**<0.001**	0.190
Axial spondyloarthritis, n=28	94.5 (84.5–96.8)	0.599	0.810	6.2 (4.2–6.8)	**0.001**	0.062
Systemic lupus erythematosus, n=33	95.3 (90.3–96.7)	**0.001**	0.215	6.3 (3.9–7.0)	**<0.001**	0.901
Systemic sclerosis, n=12	96.5 (93.7–96.7)	0.928	0.314	6.2 (5.2–6.6)	0.527	0.442
Primary Sjögren's syndrome, n=9	95.9 (88.6–96.4)	0.335	0.373	6.2 (6.0, 6.7)	–	–
Myositis, n=7	34.8 (7.7–96.2)	**<0.001**	0.571	0.2 (0, 6.3)	**<0.001**	0.477
ANCA-associated vasculitides, n=17	11.6 (2.9, 46.1)	**<0.001**	0.758	0 (0, 0.6)	**<0.001**	0.580
Polymyalgia rheumatica/giant cell arteritis, n=10	95.3 (86.7, 96.4)	0.586	0.659	5.3 (4.5, 6.5)	0.069	0.148
IgG_4_-related disease, n=7	94.6 (10.9, 95.8)	**0.011**	0.587	5.5 (0, 6.2)	**0.002**	0.636
Autoinflammatory syndromes, n=6	96.5 (82.8, 97.0)	0.567	0.593	6.5 (5.1, 6.9)	0.491	0.399
Other AIRD, n=6*	91.2 (0, 94.1)	**<0.001**	0.226	4 (0, 5.3)	**<0.001**	0.116

P values were estimated by a Wald test as combined p value of the two-part model. Statistically significant results in bold. A hyphen indicates that calculation was not possible within the model.

Adjusted multivariable analysis includes the covariates age, sex, BMI, type of vaccination, vaccine interval in days, interval between second vaccination and antibody testing in days, rheumatic diagnosis, comorbidity and immunosuppressive therapy.

*Cogan-syndrome (n=2), peripheral spondyloarthritis (n=2), polychondritis (n=1), sarcoidosis (n=1).

AIRD, autoimmune rheumatic diseases; ANCA, antineutrophil cytoplasmic antibody; BMI, body mass index; RBD, receptor-binding domain; ref, reference; S/CO, signal/cut-off.

Numerous diagnoses, such as antineutrophil cytoplasmic antibody-associated vasculitides, myositis or rheumatoid arthritis, were associated with a significantly decreased antibody response compared with ICs in unadjusted analysis (table 3). However, in adjusted analysis of patients with AIRD and IC, none of the individual diagnoses showed a significant impact on neutralising capacity or anti-RBD-IgG levels (table 3).

In adjusted analysis including possible confounders but excluding adjustment for intake of immunosuppressive medication, patients with AIRD showed an overall lower antibody response than IC (p<0.001). However, when type of immunosuppression was included as a cofactor in adjusted analysis, there was no significant difference between patients with AIRD and IC (p=0.452 for neutralising capacity and p=0.423 for anti-RBD-IgG levels).

Patients with AIRD under no immunosuppressive therapy (n=19) were off therapy for at least 6 months (no prior B-cell depleting therapies) and showed an antibody response similar to the IC group (neutralising capacity median 95.9% vs 96.5%, p=0.722, anti-RBD-IgG levels median 6.2 S/CO vs 6.7 S/CO, p=0.357, adjusted analysis, table 4).

**Table 4:** The impact of immunosuppressive therapy on neutralising capacity and anti-RBD-IgG levels in patients with AIRD (n=308) compared with controls (n=296)

Immunosuppressive therapy*, n	Neutralising capacity (%)			Anti-RBD-IgG (S/CO)		
	Median (IQR)	Unadjusted p value	Adjusted p value	Median (IQR)	Unadjusted p value	Adjusted p value
Immunocompetent controls, n=296	96.5 (93.5–97.1)	(ref)	(ref)	6.7 (6.3–7.1)	(ref)	(ref)
AIRD without immunosuppression, n=19	95.9 (94.0–96.7)	0.722	0.722	6.2 (5.3–6.9)	0.301	0.357
Anti-IL-1 mono, n=3	95.3 (45.4–96.6)	0.381	–	6.1 (2.1–6.8)	0.207	–
Anti-IL-17 mono, n=14	96.0 (89.0–96.5)	0.767	0.629	6.6 (4.4–6.9)	**0.036**	0.757
Anti-IL-6 mono, n=14	77.3 (55.1–91.8)	**0.001**	0.073	5 (3.9–5.6)	**0.035**	0.460
Anti-TNFα mono, n=46	94.0 (88.2–95.9)	0.460	0.767	6.1 (5.1–6.8)	**0.003**	0.436
Azathioprine+others, n=18	95.1 (82.9–96.9)	**<0.001**	**0.032**	6.2 (2.3–7.2)	**<0.001**	**0.008**
CYC+others, n=5	89.2 (48.0–95.4)	0.083	0.221	5.5 (2.2–6.3)	**0.040**	0.154
GC mono, n=9	95.5 (93.7–96.3)	0.717	0.563	6 (5–7.1)	0.607	0.427
HCQ mono, n=11	95.7 (95.3–96.7)	0.751	0.957	6.5 (6–7.1)	–	–
JAK inhibitors mono, n=21	71.1 (52.4–91.2)	**<0.001**	**0.006**	3.2 (2.3–6.3)	**<0.001**	**0.006**
Leflunomide+others, n=8	94.6 (87.1–96.2)	0.648	0.641	6.1 (5.7–7)	0.056	**0.041**
MMF+others, n=10	74.9 (32.1–96.6)	**<0.001**	**0.002**	4.6 (0.2–6.2)	**<0.001**	**0.004**
MTX mono, n=32	87.3 (43.1–96.2)	**<0.001**	0.113	5.4 (0.5–6.7)	**<0.001**	**0.005**
MTX+JAKi, n=6	77.5 (54.5–95.8)	**0.032**	0.111	4.6 (1.7–5.8)	**0.004**	0.058
MTX+others, n=25	79.2 (61.6–93.5)	**<0.001**	0.081	5.4 (1.6–6.7)	**<0.001**	**0.006**
RTX ≤6 months before vacc., n=23	3.7 (0–9.6)	**<0.001**	**0.001**	0 (0–0)	**<0.001**	**<0.001**
RTX >6 months before vacc., n=17	20.3 (7.0–74.0)	**<0.001**	**0.002**	0 (0–2.1)	**<0.001**	**<0.001**
Sulfasalazine+others, n=3	78.5 (76.9–96.9)	0.198	0.167	5.3 (2.8–7.0)	0.315	0.685
Others, n=13	93.1 (27.3–96.3)	**<0.001**	**0.029**	5.6 (0.6–6.9)	**<0.001**	**0.003**
No consistent therapy, n=11	94.5 (53.8–96.7)	–	–	5.9 (2.5–6.6)	–	–

P values were estimated by a Wald test as combined p value of the two-part model. Statistically significant results in bold. A hyphen indicates that calculation was not possible within the model.

Adjusted multivariable analysis includes the covariates age, sex, BMI, type of vaccination, vaccine interval in days, interval between second vaccination and antibody testing in days, rheumatic diagnosis, comorbidity and immunosuppressive therapy.

*Composition of combination therapy groups is given in online supplemental table 1.

BMI, body mass index; CYC, cyclophosphamide; GC, glucocorticoid; HCQ, hydroxychloroquine; IL, interleukin; JAK, Janus kinase; MMF, mycophenolate; mono, monotherapy; MTX, methotrexate; ref, reference; RTX, rituximab; S/CO, signal/cut-off; TNF, tumour necrosis factor.

Grouping individual diagnoses into larger categories (online supplemental table 2) or restricting the analysis to the largest patient subgroups with comparable treatments (MTX monotherapy or combination therapy or anti-TNFα monotherapy, online supplemental table 3) similarly did not reveal an influence of the diagnosis on antibody responses in multivariable analysis.

### Influence of immunosuppressive therapy on antibody response

Out of 308 patients with AIRD, 289 patients were taking immunosuppressive medications at the time of vaccination. Among these, 94 patients took conventional synthetic disease-modifying antirheumatic drugs (csDMARDs) including glucocorticoid monotherapy, 155 patients took biological DMARDs with or without csDMARDs, 29 patients took targeted synthetic DMARDs (ie, Janus kinase (JAK) inhibitors) with or without csDMARDs, and 11 patients changed their medication between vaccinations. With regard to individual therapies, the largest groups consisted of patients with anti-TNFα monotherapy (n=46) and MTX (monotherapy n=32, in combination n=31; table 4).

Within broad DMARD categories, all but biological DMARDs (excluding RTX) were associated with a statistically significant decrease in neutralising capacity and anti-RBD-IgG levels (online supplemental table 4), although median neutralising capacity remained above 90% in the csDMARD group (including glucocorticoid monotherapy) and above 70% in all other DMARD groups except RTX.

**Figure 3:**
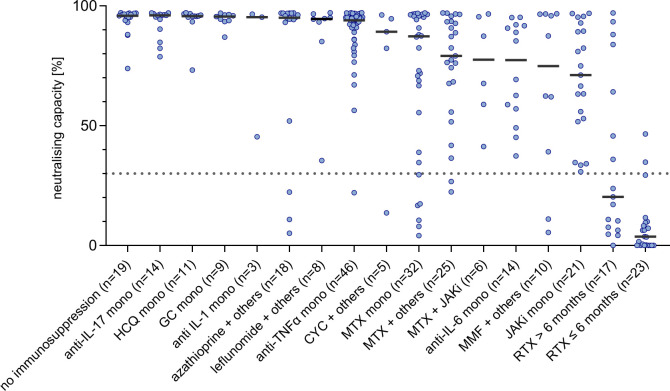
Neutralising capacity after second COVID-19 vaccination in patients with AIRD, differentiated by immunosuppressive medication. Composition of combination therapy groups is given in online supplemental table 1. Dotted line marks the cut-off value for positivity following manufacturer’s protocol (≥30%). AIRD, autoimmune rheumatic diseases; CYC, cyclophosphamide; GC, glucocorticoid; HCQ, hydroxychloroquine; JAKi, Janus kinase inhibitor; IL, interleukin; MMF, mycophenolate; mono, monotherapy; MTX, methotrexate; RTX ≤6 months, rituximab given ≤6 months prior to vaccination, RTX >6 months, rituximab given >6 months prior to vaccination; TNF, tumour necrosis factor.

Within individual therapies, patients taking intravenous cyclophosphamide (CYC, n=5, median time between last CYC dose and first vaccination 16 days, range 7–62 days; 4 patients also had one dose of CYC between vaccinations), MTX in monotherapy or combination (n=63, median weekly dose 15.0 mg), anti-IL-6 monotherapy (n=14), MMF (n=10), JAK inhibitor monotherapy (n=21) or RTX (n=40) had a reduced median neutralising capacity below 90% (table 4). Neutralising capacities differentiated by type of immunosuppressive therapy are visualised in figure 3 (anti-RBD-IgG levels: online supplemental figure 6).

In adjusted analysis, using the IC group as reference, azathioprine (n=18, median daily dose 1.3 mg/kg body weight, IQR 1–1.8 mg/kg), JAK inhibitors (n=21), MMF (n=10) and RTX (n=40) were associated with significantly lower neutralising capacities and anti-RBD-IgG levels (table 4). Leflunomide+others (n=8), MTX monotherapy (n=32) and MTX+others (n=25) resulted in significantly lower results in anti-RBD-IgG levels, but not in neutralising capacity (adjusted analysis, table 4).

Glucocorticoid monotherapy (median daily dose 3.75 mg, IQR 2.5–5 mg) did not have a significant effect on antibody levels (table 4). The same was true for the intake of low-dose prednisolone (defined as ≤5 mg/day, n=71 patients, median daily dose 5.0 mg, IQR 2.0–5.0 mg, see online supplemental table 1) in addition to any of the other therapies in adjusted analysis within patients with AIRD (neutralising capacity p=0.062; anti-RBD-IgG levels p=0.331).

### Severe impact of RTX on antibody response

**Figure 4:**
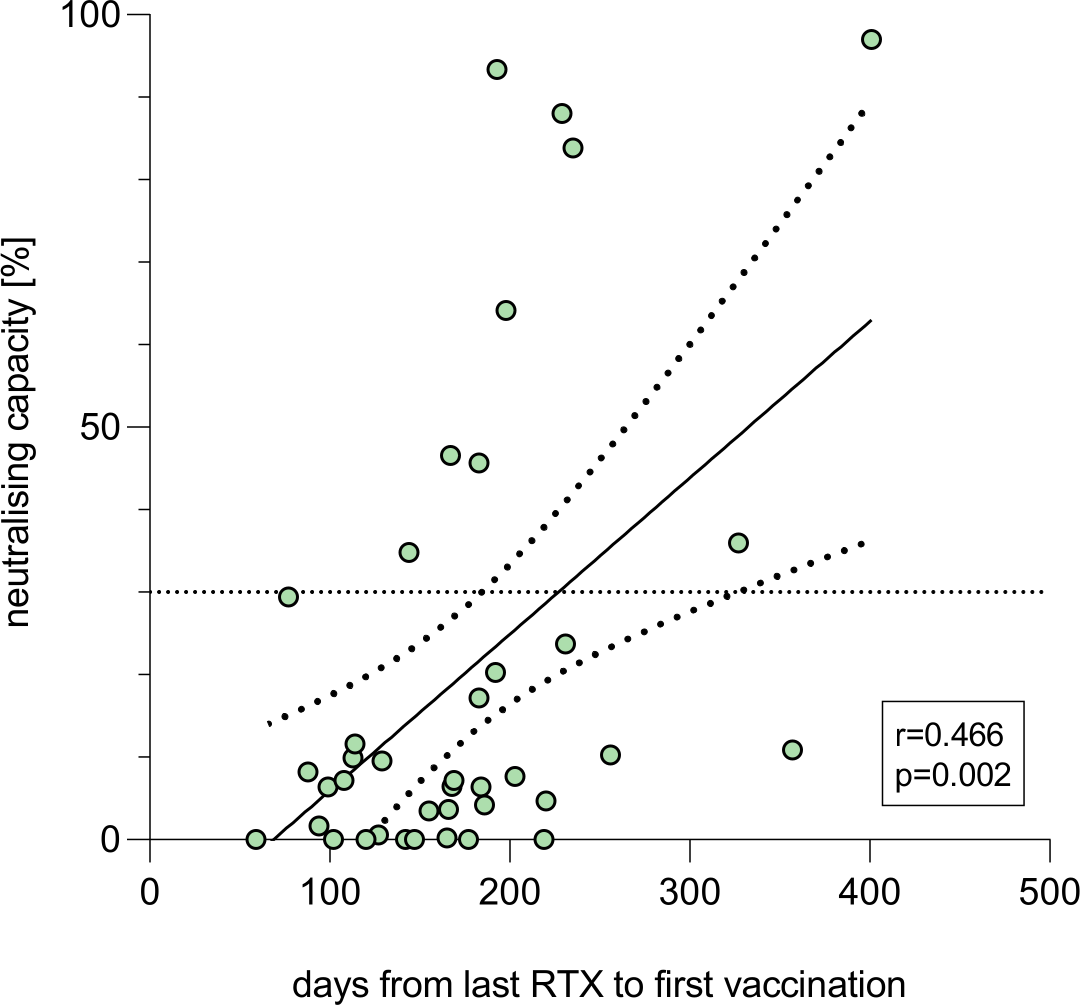
Patients with AIRD under rituximab (RTX) therapy (n=40). r and p according to Spearman’s rank correlation between days from last RTX infusion to first vaccination and neutralising capacity after second COVID-19 vaccination. Dotted line marks the cut-off value for positivity following manufacturer’s protocol (≥30%).

Patients receiving RTX therapy (n=40) showed severely reduced antibody responses in comparison to patients on other medication or to ICs (neutralising capacity median 7.43%, anti-RBD-IgG median 0.0 S/CO); 77.5% (n=31) of all RTX patients had a negative vaccination response (neutralising capacity <30%). Median time from last RTX infusion to first COVID-19 vaccination was 168 days (IQR 122–202 days). Patients who had received their last RTX dose 6 months or less before vaccination had the lowest neutralising capacity (median 3.67%) and anti-RBD-IgG levels (median 0 S/CO) of all patients. A longer time span between the last administration of RTX and the first dose of vaccination correlated with a higher neutralising capacity (Spearman’s rank correlation, r=0.466, p=0.002, figure 4) and anti-RBD-IgG levels (Spearman’s rank correlation, r=0.364, p=0.021, online supplemental figure 7).

### Influence of therapy hold on antibody response

Out of 308 patients with AIRD, 69 reported to have held their medication for at least one vaccination. The largest groups were formed by MTX monotherapy patients (n=14) and anti-TNFα monotherapy patients (n=12). The median treatment delay of both vaccinations combined, defined as the actual medication interval around the vaccinations minus the regular intake interval, was 14 days (IQR 6.25–28) for MTX and 18.5 days (IQR 13.25–33.25) for anti-TNFα monotherapy treatment.

Patients who held MTX for at least one vaccination (n=14) showed a 25.9 percentage points higher neutralising capacity than patients who continued their MTX intake (n=18, 95.7% vs 69.8%, p=0.043). This effect was also seen in the anti-RBD-IgG concentration (6.4 vs 3.6 S/CO, p=0.021).

Patients who held their anti-TNFα medication (n=12) showed no significant differences in neutralising capacity when compared with patients who continued anti-TNFα intake during both vaccinations (n=34, 95.1% vs 93.9%, p=0.812). The same was true for anti-RBD-IgG levels (6.4 vs 5.8 S/CO, p=0.370).

## Discussion

This study found a lower antibody response after 2 doses of COVID-19 vaccinations among patients with rheumatic diseases than among ICs, in line with published research.^5 6 13^ We found a slightly lower positivity rate among patients with AIRD (83.8% for neutralising capacity and 79.9% for anti-RBD-IgG levels) than others (Furer *et al*: 86.0%,^13^ Deepak *et al*: 88.7%^6^). This is probably due to varying compositions of the observed cohorts.

While certain diagnoses were individually associated with lower antibody responses, this could not be confirmed in adjusted analysis, which suggests that the presumed effect of the diagnoses is confounded by the respective therapies. Accordingly, in adjusted comparison of all patients with AIRD versus IC, a significant difference in antibody results was no longer observed when type of immunosuppressive therapy was included as a cofactor. Moreover, the group of 19 patients with AIRD without any therapeutic immunosuppression showed similar antibody responses to the IC group in our analysis, further emphasising the effect of treatment rather than diagnosis. Our findings indicate that the lower antibody response in patients with AIRD can generally be attributed to immunosuppressive therapies, and not to the rheumatic diagnosis, as previously suggested by Simon *et al*.^5^ In line with our findings, another group reported no significant associations of broad disease categories with responder rates, although without a control group,^9^ while Furer *et al* reported lower responder rates in patients with certain diagnoses, but also argued that these findings were at least partially explained by immunosuppressive therapy.^13^

Many antirheumatic therapies (eg, HCQ or biological DMARDs including monoclonal antibodies against IL-1, IL-17 or TNFα) were not associated with a negative effect on vaccination response in our cohort. However, some showed mildly to moderately reduced median antibody levels (eg, csDMARDs like MTX and MMF or JAK inhibitors), while the most severely reduced antibody results compared with all other patients and controls were seen in RTX patients. The duration between the last RTX infusion and the first COVID-19 vaccination correlated with vaccination response in these patients, emphasising the importance of adequate scheduling of vaccination for RTX patients. These results are in line with previous research.^8 11 13^ Although small, our group of CYC patients did not show a significantly impaired humoral immune response in comparison to the IC. The effect of CYC on vaccination response has not yet been sufficiently investigated, but a previous cohort confirmed a stronger influence of RTX compared with CYC.^24^ As patients could benefit from treatment alternatives to RTX during the pandemic,^25^ CYC might be considered in certain indications.

The effect of MTX monotherapy or combination therapy on antibody response was statistically significant for anti-RBD-IgG levels but not for neutralisation capacity. However, patients who paused MTX were not excluded in this analysis. We and other groups have already described lower antibody responses in patients continuing MTX and the benefit of pausing MTX during vaccination.^16 26^ Unlike MTX hold, pausing anti-TNFα therapy had no influence on immune response in our cohort. The negative effect of MMF has been described previously^7 13^ and can be confirmed with our data. Patients treated with JAK inhibitors also showed a lower antibody response after COVID-19 vaccination in our cohort. The same observation was made by Iancovici *et al*,^12^ while other groups focusing on responder rates reported no impaired immune response under JAK inhibitor therapy.^7 13^ Seror *et al* observed an increased rate of non-responders under upadacitinib therapy despite an overall high response rate under JAK inhibitor therapy.^27^ A significant antibody reduction regardless of responder status seems possible under JAK inhibitor therapy and an effect of therapy pause should be investigated further.

Patients taking azathioprine showed significantly reduced antibody responses in statistical analysis in our cohort despite high median response rates, which may be attributed to outliers and should be interpreted with caution. In previous studies, azathioprine did not have a negative impact on antibody response, however, patients on azathioprine therapy were often analysed together with other therapeutic groups and case numbers of azathioprine patients were low.^6 28^ The existing overall data do not allow to draw definitive conclusions regarding the effect of azathioprine.

Medication with prednisolone, either as monotherapy (n=9, median daily dose 3.75 mg) or in addition to other immunosuppressive therapies (n=71, median and maximum daily dose 5.0 mg) did not lead to a statistically significant reduction of antibody levels in our cohort, whereas others have reported a negative effect of glucocorticoid therapy.^6 13^ Notably, dosage was higher in both these cohorts, so the negative impact of glucocorticoids may be dose-dependent. A negative effect of abatacept therapy has also been described previously,^13^ but could not be analysed in our cohort because of a low abatacept case number.

In addition to type of immunosuppressive therapy, we found evidence that age, type of vaccination and time interval between vaccinations may have an additional impact on the antibody response. Old age is already known to be associated with lower antibody responses.^19 29^ Our data also confirm substantially lower antibody responses after two doses of AZD1222 compared with other vaccination regimens with at least one dose of an mRNA vaccine in patients with AIRD.^24 30^ The positive effect of a longer vaccine interval on humoral immune response is in line with previously published work,^31 32^ but has to our knowledge not been described among patients with AIRD yet.

A strength of this study is the size of the study population and the control group, which allowed adjustment for possible confounders by using multivariable analysis. Therefore, differences in variables between the control group and the AIRD group, such as age or vaccine interval, could be addressed by inclusion into the model. Large data overlaps between IC and AIRD with regard to these variables allowed effective adjustment by multivariable analysis. Furthermore, the variety and size of the study population allowed differentiation into individual diagnosis and therapy groups in addition to a supplementary investigation with broader categories, which was the sole analysis in some previous studies.^6 30^ The potential bias caused by group inhomogeneity was thereby reduced, but this also led to smaller case numbers, which may be associated with a lesser accuracy of the results.

The statistical analysis was designed to allow examination of relevant differences beyond the responder status alone while avoiding overestimation of small and clinically meaningless differences between groups of very high responders, which also overcomes potentially lower accuracy of the test systems in the high range of values. The use of two independent tests to assess humoral immunogenicity served as an internal validation of our data. Different approaches in test methodology may have led to small differences, but generally both tests yielded similar results.

This study has limitations. Despite employing adjusted multivariable analysis, residual confounding cannot be excluded with certainty. Patients with AIRD and IC showed a remarkable difference in age, but the age distributions within the two groups had a sufficient overlap and the adjustment of age in multivariable analyses yielded valid estimates. As this was an exploratory data analysis, we did not account for multiple testing. Disease activity or flare rates were not routinely assessed. An induction of disease flares or a possible impact of a higher disease activity on antibody response can therefore not be excluded. The measurement of T-cell responses was not part of the study design. However, humoral vaccination response has been shown to be an adequate means to determine vaccine immunogenicity^15^ and higher antibody levels correlate with a better clinical outcome.^14 33^ Antibody levels and surrogate neutralisation results were tested against Wuhan antigens and may overestimate protection against infection with omicron sublineages of SARS-CoV-2,^34 35^ but risk reduction with regard to severe courses of COVID-19 remains intact.^36^

In conclusion, the results of this study indicate that AIRD diagnosis itself does not cause lower antibody responses after COVID-19 vaccination. While RTX most severely impacts humoral immunogenicity, a few other therapies like MMF, MTX and JAK inhibitors also have negative influences on antibody levels. For some individual therapies (ie, CYC, anti-IL-6, azathioprine and JAK inhibitors), even larger dedicated cohorts or meta-analyses are needed to reliably quantify the effects of continued or interrupted treatment on vaccination. We advocate antibody testing in patients with AIRD who are at risk of decreased antibody responses due to certain immunosuppressive treatments. Older age (≥60 years), vaccination with two doses of AZD1222 and short vaccination intervals also should be taken into account. Booster vaccinations are especially important in these patients with potentially impaired immune responses and additional booster vaccinations should be considered.

## Data Availability

Data are available on reasonable request. All data relevant to the study are included in the article or uploaded as online supplemental information.

## References

[1] Grainger R, Kim AHJ, Conway R, et al. COVID-19 in people with rheumatic diseases: risks, outcomes, treatment considerations. Nat Rev Rheumatol 2022;18:191–204. 10.1038/s41584-022-00755-x 35217850 PMC8874732

[2] Arnold J, Winthrop K, Emery P. COVID-19 vaccination and antirheumatic therapy. Rheumatology 2021;60:3496–502. 10.1093/rheumatology/keab223 33710296 PMC7989162

[3] Sun J, Zheng Q, Madhira V, et al. Association between immune dysfunction and COVID-19 breakthrough infection after SARS-CoV-2 vaccination in the US. JAMA Intern Med 2022;182:153–62. 10.1001/jamainternmed.2021.7024 34962505 PMC8715386

[4] Geisen UM, Berner DK, Tran F, et al. Immunogenicity and safety of anti-SARS-CoV-2 mRNA vaccines in patients with chronic inflammatory conditions and immunosuppressive therapy in a monocentric cohort. Ann Rheum Dis 2021;80:1306–11. 10.1136/annrheumdis-2021-220272 33762264 PMC8117443

[5] Simon D, Tascilar K, Fagni F, et al. SARS-CoV-2 vaccination responses in untreated, conventionally treated and anticytokine-treated patients with immune-mediated inflammatory diseases. Ann Rheum Dis 2021;80:1312–6. 10.1136/annrheumdis-2021-220461 33958324 PMC8103562

[6] Deepak P, Kim W, Paley MA, et al. Effect of Immunosuppression on the Immunogenicity of mRNA Vaccines to SARS-CoV-2 : A Prospective Cohort Study. Ann Intern Med 2021;174:1572–85. 10.7326/M21-1757 34461029 PMC8407518

[7] Ruddy JA, Connolly CM, Boyarsky BJ, et al. High antibody response to two-dose SARS-CoV-2 messenger RNA vaccination in patients with rheumatic and musculoskeletal diseases. Ann Rheum Dis 2021;80:1351–2. 10.1136/annrheumdis-2021-220656 34031032 PMC8843949

[8] Spiera R, Jinich S, Jannat-Khah D. Rituximab, but not other antirheumatic therapies, is associated with impaired serological response to SARS- CoV-2 vaccination in patients with rheumatic diseases. Ann Rheum Dis 2021;80:1357–9. 10.1136/annrheumdis-2021-220604 33975857

[9] Braun-Moscovici Y, Kaplan M, Braun M, et al. Disease activity and humoral response in patients with inflammatory rheumatic diseases after two doses of the Pfizer mRNA vaccine against SARS-CoV-2. Ann Rheum Dis 2021;80:1317–21. 10.1136/annrheumdis-2021-220503 34144967

[10] Rubbert-Roth A, Vuilleumier N, Ludewig B, et al. Anti-SARS-CoV-2 mRNA vaccine in patients with rheumatoid arthritis. The Lancet Rheumatology 2021;3:e470–2. 10.1016/S2665-9913(21)00186-7 34124693 PMC8186851

[11] Moor MB, Suter-Riniker F, Horn MP, et al. Humoral and cellular responses to mRNA vaccines against SARS-CoV-2 in patients with a history of CD20 B-cell-depleting therapy (RituxiVac): an investigator-initiated, single-centre, open-label study. The Lancet Rheumatology 2021;3:e789–97. 10.1016/S2665-9913(21)00251-4 34514436 PMC8423431

[12] Iancovici L, Khateeb D, Harel O, et al. Rheumatoid arthritis patients treated with Janus kinase inhibitors show reduced humoral immune responses following BNT162b2 vaccination. Rheumatology 2022;61:keab879:3439–47. 10.1093/rheumatology/keab879 34849628 PMC8767876

[13] Furer V, Eviatar T, Zisman D, et al. Immunogenicity and safety of the BNT162b2 mRNA COVID-19 vaccine in adult patients with autoimmune inflammatory rheumatic diseases and in the general population: a multicentre study. Ann Rheum Dis 2021;80:1330–8. 10.1136/annrheumdis-2021-220647 34127481

[14] Feng S, Phillips DJ, White T, et al. Correlates of protection against symptomatic and asymptomatic SARS-CoV-2 infection. Nat Med 2021;27:2032–40. 10.1038/s41591-021-01540-1 34588689 PMC8604724

[15] Khoury DS, Cromer D, Reynaldi A, et al. Neutralizing antibody levels are highly predictive of immune protection from symptomatic SARS-CoV-2 infection. Nat Med 2021;27:1205–11. 10.1038/s41591-021-01377-8 34002089

[16] Arumahandi de Silva AN, Frommert LM, Albach FN, et al. Pausing methotrexate improves immunogenicity of COVID-19 vaccination in elderly patients with rheumatic diseases. Ann Rheum Dis 2022;81:881–8. 10.1136/annrheumdis-2021-221876 35288376 PMC9120396

[17] Habermann E, Gieselmann L, Tober-Lau P, et al. Pausing methotrexate prevents impairment of omicron BA.1 and BA.2 neutralisation after COVID-19 booster vaccination. RMD Open 2022;8:e002639. 10.1136/rmdopen-2022-002639 36216410 PMC9556747

[18] Specker C, Aries P, Braun J, et al. Aktualisierte Handlungsempfehlungen der Deutschen Gesellschaft für Rheumatologie für die Betreuung von Patienten mit entzündlich-rheumatischen Erkrankungen im Rahmen der SARS-CoV‑2/COVID‑19-Pandemie einschließlich Empfehlungen zur COVID‑19-Impfung. Z Rheumatol 2021;80:570–87. 10.1007/s00393-021-01056-6 34309739 PMC8311067

[19] Schwarz T, Tober-Lau P, Hillus D, et al. Delayed antibody and T-cell response to BNT162b2 vaccination in the elderly, Germany. Emerg Infect Dis 2021;27:2174–8. 10.3201/eid2708.211145 34102097 PMC8314803

[20] Hillus D, Schwarz T, Tober-Lau P, et al. Safety, reactogenicity, and immunogenicity of homologous and heterologous prime-boost immunisation with ChAdOx1 nCoV-19 and BNT162b2: a prospective cohort study. The Lancet Respiratory Medicine 2021;9:1255–65. 10.1016/S2213-2600(21)00357-X 34391547 PMC8360702

[21] Tan CW, Chia WN, Qin X, et al. A SARS-CoV-2 surrogate virus neutralization test based on antibody-mediated blockage of ACE2–spike protein–protein interaction. Nat Biotechnol 2020;38:1073–8. 10.1038/s41587-020-0631-z 32704169

[22] Cragg JG. Some statistical models for limited dependent variables with application to the demand for durable goods. Econometrica 1971;39:829–44. 10.2307/1909582

[23] Fox J. Describing univariate distributions. In: Modern methods of data analysis, 1990: 58–125.

[24] Prendecki M, Clarke C, Edwards H, et al. Humoral and T-cell responses to SARS-CoV-2 vaccination in patients receiving immunosuppression. Ann Rheum Dis 2021;80:1322–9. 10.1136/annrheumdis-2021-220626 34362747 PMC8350975

[25] Boekel L, Wolbink GJ. Rituximab during the COVID-19 pandemic: time to discuss treatment options with patients. Lancet Rheumatol 2022;4:e154–5. 10.1016/S2665-9913(21)00418-5 34977601 PMC8700274

[26] Araujo CSR, Medeiros-Ribeiro AC, Saad CGS, et al. Two-Week methotrexate discontinuation in patients with rheumatoid arthritis vaccinated with inactivated SARS-CoV-2 vaccine: a randomised clinical trial. Ann Rheum Dis 2022;81:889–97. 10.1136/annrheumdis-2021-221916 35193873

[27] Seror R, Camus M, Salmon J-H, et al. Do JAK inhibitors affect immune response to COVID-19 vaccination? data from the MAJIK-SFR registry. The Lancet Rheumatology 2022;4:e8–11. 10.1016/S2665-9913(21)00314-3 34642669 PMC8494471

[28] Boyarsky BJ, Ruddy JA, Connolly CM, et al. Antibody response to a single dose of SARS-CoV-2 mRNA vaccine in patients with rheumatic and musculoskeletal diseases. Ann Rheum Dis 2021;80:1098–9. 10.1136/annrheumdis-2021-220289 33757968 PMC8822300

[29] Müller L, Andrée M, Moskorz W, et al. Age-Dependent immune response to the Biontech/Pfizer BNT162b2 coronavirus disease 2019 vaccination. Clin Infect Dis 2021;73:2065–72. 10.1093/cid/ciab381 33906236 PMC8135422

[30] Tran AP, Tassone D, Nossent J, et al. Antibody response to the COVID-19 ChAdOx1nCov-19 and BNT162b vaccines after temporary suspension of DMARD therapy in immune-mediated inflammatory disease (RESCUE). RMD Open 2022;8:e002301. 10.1136/rmdopen-2022-002301 35577478 PMC9114315

[31] Payne RP, Longet S, Austin JA, et al. Immunogenicity of standard and extended dosing intervals of BNT162b2 mRNA vaccine. Cell 2021;184:5699–714. 10.1016/j.cell.2021.10.011 34735795 PMC8519781

[32] Grunau B, Goldfarb DM, Asamoah-Boaheng M, et al. Immunogenicity of extended mRNA SARS-CoV-2 vaccine dosing intervals. JAMA 2022;327:279–81. 10.1001/jama.2021.21921 34860253 PMC8642809

[33] Gilbert PB, Montefiori DC, McDermott AB, et al. Immune correlates analysis of the mRNA-1273 COVID-19 vaccine efficacy clinical trial. Science 2022;375:43–50. 10.1126/science.abm3425 34812653 PMC9017870

[34] Willett BJ, Grove J, MacLean OA, et al. SARS-CoV-2 omicron is an immune escape variant with an altered cell entry pathway. Nat Microbiol 2022;7:1161–79. 10.1038/s41564-022-01143-7 35798890 PMC9352574

[35] Hoffmann M, Krüger N, Schulz S, et al. The omicron variant is highly resistant against antibody-mediated neutralization: implications for control of the COVID-19 pandemic. Cell 2022;185:447–56. 10.1016/j.cell.2021.12.032 35026151 PMC8702401

[36] Collie S, Champion J, Moultrie H, et al. Effectiveness of BNT162b2 vaccine against omicron variant in South Africa. N Engl J Med 2022;386:494–6. 10.1056/NEJMc2119270 34965358 PMC8757569

