# Supplementary material for: Type of vaccine and immunosuppressive therapy but not diagnosis critically influence antibody response after COVID-19 vaccination in patients with rheumatic disease
Source: RMD Open. 2022 Dec 6;8(2):e002650. doi: 10.1136/rmdopen-2022-002650 (PMC9729845; doi:10.1136/rmdopen-2022-002650)
Supplement: Supplementary data [file rmdopen-2022-002650supp001.pdf]

Supplemental material

Supplemental table 1: Composition of combination therapy groups

Name of therapy group	Composition
Anti-IL-6 mono, n=14	*additional low dose prednisolone n=1*
Anti-TNFα mono, n=46	*additional low dose prednisolone n=3*
Azathioprine+others, n=18	Azathioprine monotherapy (n=8)<br>Azathioprine + anti-TNFα (n=1), HCQ (n=8), prednisolone 15mg/day (n=1)<br>*additional low dose prednisolone n=8*
CYC+others, n=5	CYC monotherapy (n=3)<br>CYC + prednisolone 10mg/day (n=2)<br>*additional low dose prednisolone n=1*
HCQ mono, n=11	*additional low dose prednisolone n=1*
JAK inhibitors mono, n=21	*additional low dose prednisolone n=4*
Leflunomide+others, n=8	Leflunomide monotherapy (n=7)<br>Leflunomide + anti-TNFα (n=1)<br>*additional low dose prednisolone n=5*
MMF+others, n=10	MMF monotherapy (n=3)<br>MMF + HCQ (n=4), belimumab (n=1), HCQ and belimumab (n=2)<br>*additional low dose prednisolone n=8*
MTX mono, n=32	*additional low dose prednisolone n=11*
MTX+JAKi, n=6	*additional low dose prednisolone n=2*
MTX+others, n=25	MTX + anti-TNFα (n=17), anti-IL-12/23 (n=1), anti-IL-17 (n=2), HCQ (n=2), leflunomide (n=3)<br>*additional low dose prednisolone n=7*
No consistent therapy, n=11	Treatment change between vaccinations
Others, n=13	Anti-IL12/23 (n=2), anti-IL12/23 + azathioprine (n=1), MTX + anti-IL-6 (n=2), abatacept + MTX (n=1), abatacept + sulfasalazin (n=1), belimumab + HCQ (n=1), JAK inhibitor + prednisolon > 5 mg/d (n=2), ≥ 2 different DMARDs + prednisolone > 5 mg/d (n=3)<br>*additional low dose prednisolone n=2*
RTX > 6 months, n=17	RTX monotherapy (n=12)<br>RTX + MTX (n=4), prednisolone 10mg/day (n=1)<br>*additional low dose prednisolone n=10*
RTX ≤ 6 months, n=23	RTX monotherapy (n=13)<br>RTX + MTX (n=8), azathioprine (n=1), JAKi (n=1)<br>*additional low dose prednisolone n=8*
Sulfasalazine+others, n=3	Sulfasalazine monotherapy (n=1)<br>Sulfasalazine + anti-TNFα (n=1), anti-IL-17 (n=1)

CYC, Cyclophosphamide; GC, glucocorticoid; HCQ, hydroxychloroquine; JAK, Janus kinase; IL, interleukin; MMF, mycophenolate; MTX, methotrexate; RTX ≤ 6 months, rituximab given ≤ 6 months prior to vaccination, RTX > 6 months, rituximab given > 6 months prior to vaccination; TNF, tumour necrosis factor.

Supplemental table 2: The impact of rheumatic diagnosis categories on neutralising capacity and anti-RBD-IgG levels in AIRD patients (n=308) compared to controls (n=296)

	**Neutralising capacity [%]**			**Anti-RBD-IgG [S/CO]**		
**Rheumatic diagnosis category, n**	**Median (IQR)**	**Unadjusted p value**	**Adjusted p value**	**Median (IQR)**	**Unadjusted p value**	**Adjusted p value**
Immunocompetent controls, n=296	96.5 (93.5, 97.1)	(ref)	(ref)	6.7 (6.3, 7.1)	(ref)	(ref)
Inflammatory Joint Diseases, n=203	88.0 (55.7, 95.4)	**<0.001**	0.444	5.5 (2.2, 6.5)	**<0.001**	0.532
Connective Tissue Diseases / Myositis, n=61	95.7 (87.9, 96.7)	**<0.001**	0.548	6.1 (4.7, 6.9)	**0.001**	0.409
Vasculitis, n=29	46.6 (5.5, 94.5)	**<0.001**	0.235	1.1 (0, 5.3)	**<0.001**	0.436
Others, n=15*	95.3 (45.4, 96.4)	**<0.001**	0.415	5.9 (2.1, 6.7)	**0.001**	0.699

P values were estimated by a Wald test as combined p value of the two-part model. Statistically significant results in bold.
Adjusted multivariable analysis includes the covariates age, sex, BMI, type of vaccination, vaccine interval in days, interval between second vaccination and antibody testing in days, rheumatic diagnosis category, comorbidity and immunosuppressive therapy.
* IgG4-related disease (n=7), autoinflammatory syndromes (n=6), polychondritis (n=1), sarcoidosis (n=1)
ref, reference; S/CO, signal/cut- off.

Supplemental table 3: The impact of rheumatic diagnosis on neutralising capacity and anti-RBD-IgG levels within MTX- or anti-TNFα monotherapy treated patients

Rheumatic diagnosis, n	Neutralising capacity [%]			Anti-RBD-IgG [S/CO]		
	**Median (IQR)**	**Unadjusted p value**	**Adjusted p value**	**Median (IQR)**	**Unadjusted p value**	**Adjusted p value**
**MTX treated patients (n=63)**						
Psoriatic arthritis, n=9	93.6 (64.6, 96.9)	(ref)	(ref)	5.4 (2.5, 6.2)	(ref)	(ref)
Rheumatoid arthritis, n=42	76.9 (54.5, 93.1)	0. 803	0.899	5.3 (1.2, 6.7)	0.142	0.124
Other AIRD, n=12*	91.3 (69.2, 96.4)	0.737	0.681	5.2 (1.6, 6.2)	0.721	0.639
**Anti-TNF mono treated patients (n=46)**						
Psoriatic arthritis, n=11	95.5 (90.7, 96.8)	(ref)	(ref)	5.9 (5.5, 6.9)	(ref)	(ref)
Rheumatoid arthritis, n=18	92.3 (85.3, 95.1)	0.524	0.284	6.1 (5.1, 6.4)	0.517	0.155
Axial spondyloarthritis, n=17	94.3 (91.5, 96.6)	0.634	0.462	6.3 (4.5, 6.8)	0.346	0.971

P values were estimated by a Wald test as combined p value of the two-part model. Statistically significant results in bold.
Adjusted multivariable analysis includes the covariates age, type of vaccination and rheumatic diagnosis.
* Axial spondyloarthritis (n=3), Polymyalgia rheumatica/giant cell arteritis (n=3), myositis (n=2), Systemic sclerosis (n=2), Primary Sjögren's syndrome (n=1), ANCA-associated vasculitis (n=1).
MTX, methotrexate; ref, reference; S/CO, signal/cut- off, TNF, tumour necrosis factor.

Supplemental table 4: The impact of immunosuppressive therapy categories on neutralising capacity and anti-RBD-IgG levels in AIRD patients (n=308) compared to controls (n=296)

	**Neutralising capacity [%]**			**Anti-RBD-IgG [S/CO]**		
**Immunosuppressive therapy category, n**	**Median (IQR)**	**Unadjusted p value**	**Adjusted p value**	**Median (IQR)**	**Unadjusted p value**	**Adjusted p value**
Immunocompetent controls, n=296	96.5 (93.5, 97.1)	(ref)	(ref)	6.7 (6.3, 7.1)	(ref)	(ref)
AIRD without immunosuppression, n=19	95.9 (94.0, 96.7)	0.722	0.722	6.2 (5.3, 6.9)	0.301	0.357
RTX mono/combi, n=40	7.4 (0.8, 28.0)	**<0.001**	**<0.001**	0 (0, 0)	**<0.001**	**<0.001**
bDMARD only, n=79*	93.9 (84.8, 96)	**<0.001**	0.129	6.0 (4.6, 6.8)	**0.023**	0.107
csDMARD, n=94**	94.5 (73.2, 96.3)	**<0.001**	**0.001**	6.0 (4.5, 6.8)	**<0.001**	**0.003**
csDMARD+bDMARD, n=36*	78.3 (52.9, 93.5)	**<0.001**	**0.001**	5.2 (1.5, 6.8)	**<0.001**	**0.001**
no consistent therapy, n=11	94.5 (53.8, 96.7)	-	-	5.9 (2.5, 6.6)	-	-
tsDMARD+-csDMARD, n=29	71.1 (52.4, 94.1)	**<0.001**	**0.001**	3.9 (2.3, 6.1)	**<0.001**	**0.001**

P values were estimated by a Wald test as combined p value of the two-part model. Statistically significant results in bold.
Adjusted multivariable analysis includes the covariates age, sex, BMI, type of vaccination, vaccine interval in days, interval between second vaccination and antibody testing in days, rheumatic diagnosis, comorbidity and immunosuppressive therapy category.
* Excluding rituximab.
** Including glucocorticoid monotherapy.
bDMARD, biological disease modifying antirheumatic drugs; csDMARD, conventional synthetic disease modifying antirheumatic drugs; ref, reference; RTX, rituximab; S/CO, signal/cut- off, tsDMARD, targeted synthetic disease modifying antirheumatic drugs.

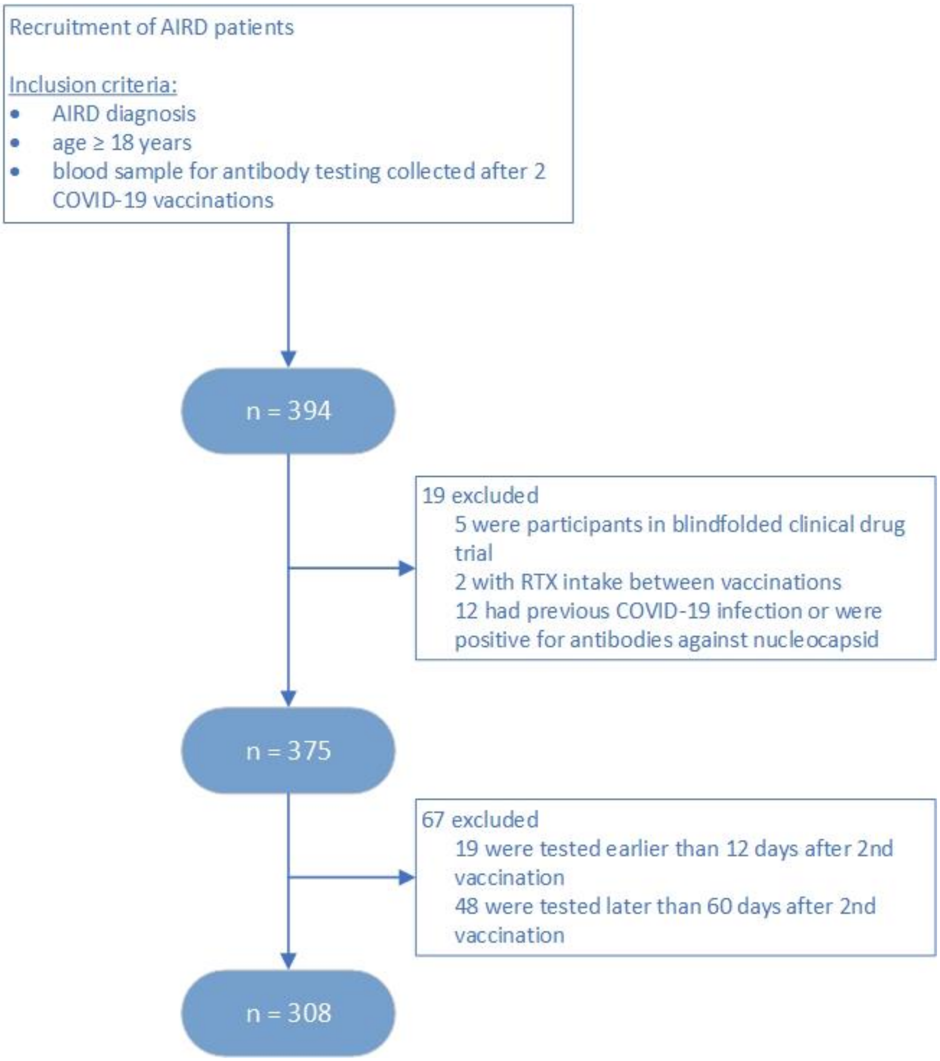

Supplemental Figure 1: Recruitment and selection of AIRD patients.

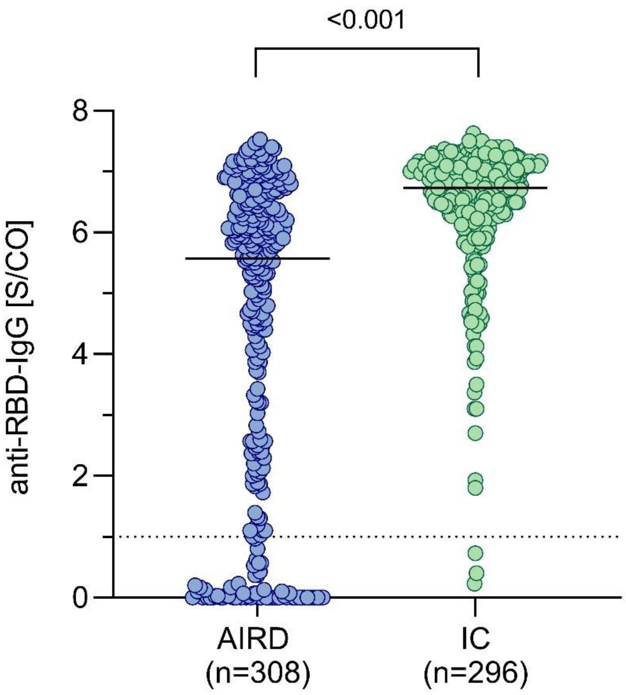

Supplemental Figure 2: Comparison of anti-RBD-IgG levels after second COVID-19 vaccination in patients with autoimmune rheumatic diseases (AIRD) and immunocompetent controls (IC). Dotted line marks the cut-off value for positivity following manufacturer's protocol (>1 S/CO). P values were estimated by a Wald test as combined p value of an unadjusted two-part model.

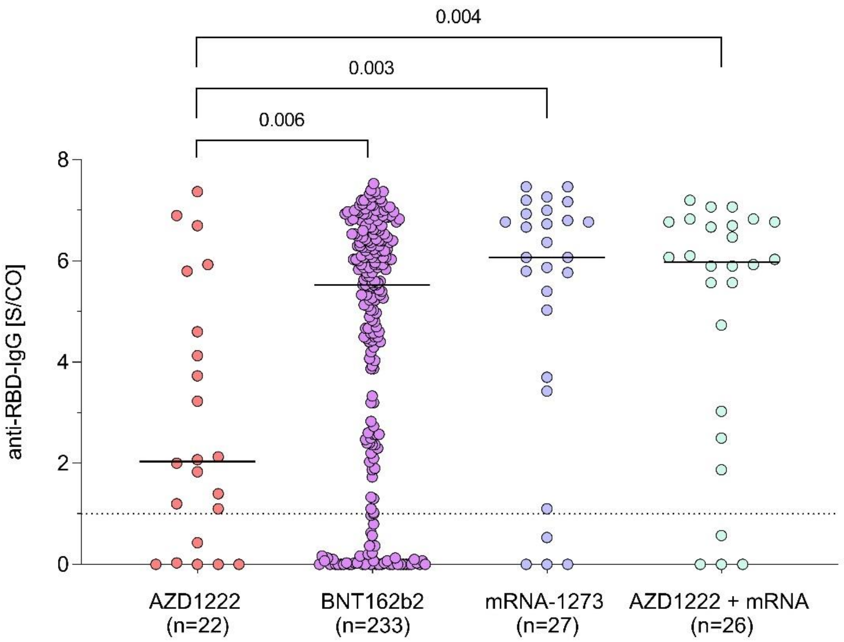

Supplemental Figure 3: Anti-RBD-IgG levels after second COVID-19 vaccination in AIRD patients, differentiated by vaccination regime. Dotted line marks the cut-off value for positivity following manufacturer's protocol (>1 S/CO). P values were estimated by a Wald test as combined p value of an unadjusted two-part model.

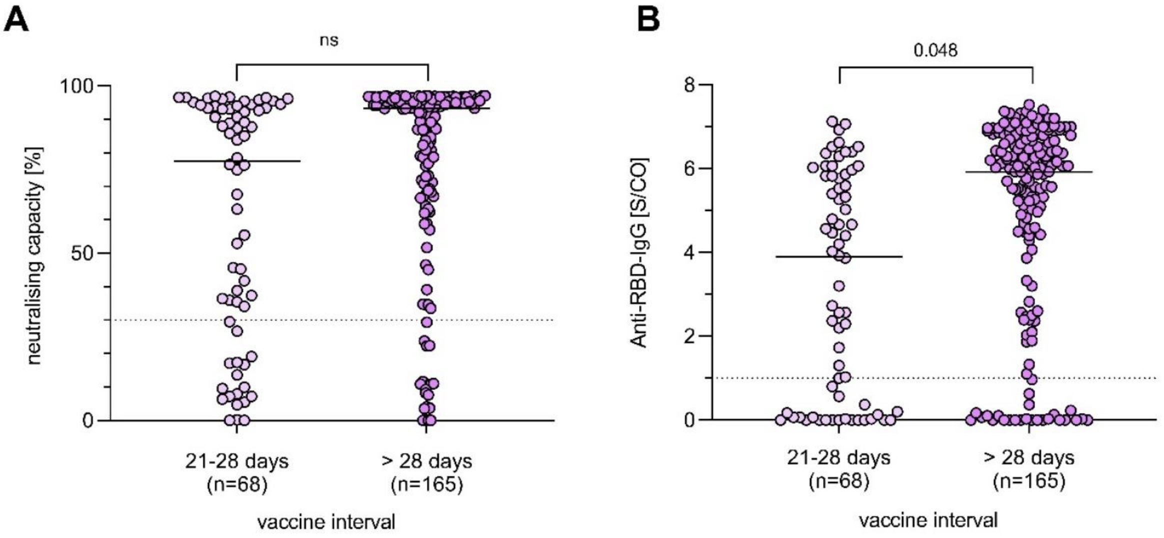

Supplemental Figure 4: Neutralising capacity (A) and anti-RBD-IgG levels (B) after second COVID-19 vaccination in AIRD patients vaccinated with BNT162b2 (n=233), differentiated by short (21-28 days) and long (>28 days) vaccine interval (time between first and second BNT162b2 vaccination). Dotted line marks the cut-off value for positivity following manufacturer's protocol (A: neutralising capacity ≥30 %; B: anti-RBD-IgG levels >1 S/CO). P values were estimated by a Wald test as combined p value of an unadjusted two-part model.

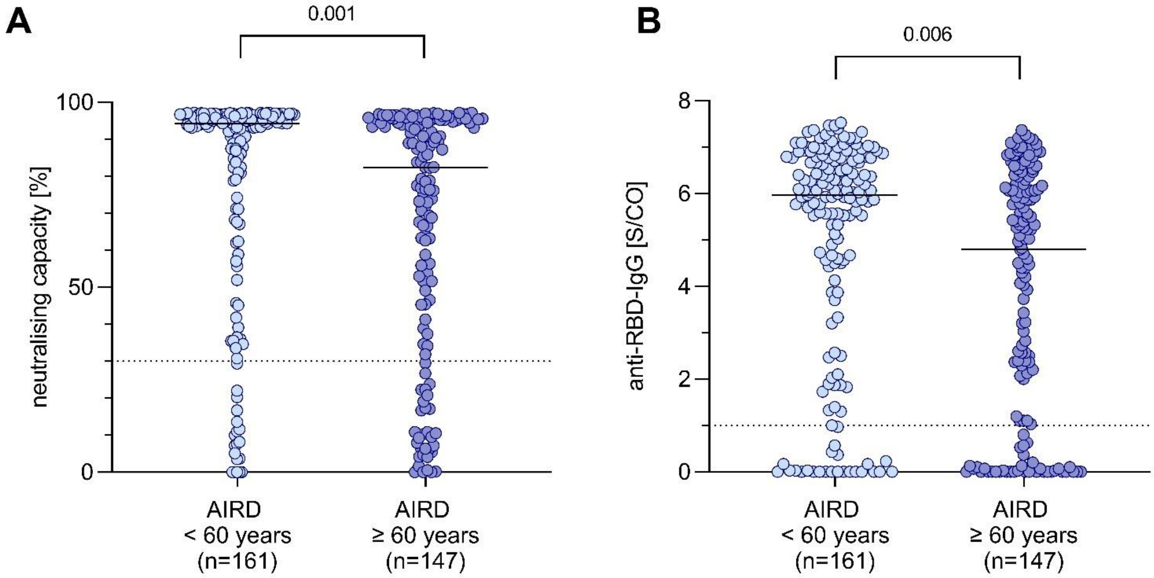

Supplemental Figure 5: Neutralising capacity (A) and anti-RBD-IgG levels (B) after second COVID-19 vaccination in AIRD patients (n=308), differentiated by young (<60 years) and old age (≥60 years) days). Dotted line marks the cut-off value for positivity following manufacturer's protocol (A: neutralising capacity ≥30 %; B: anti-RBD-IgG levels >1 S/CO). P values were estimated by a Wald test as combined p value of an unadjusted two-part model.

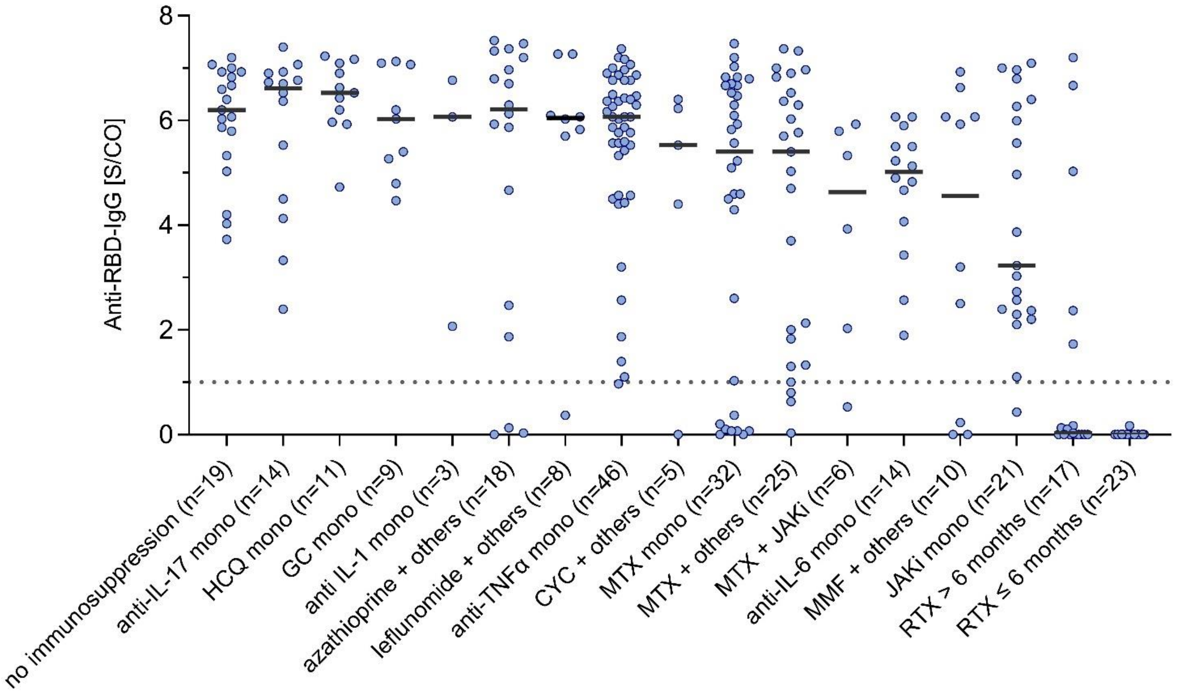

Supplemental Figure 6: Anti-RBD-IgG levels after second COVID-19 vaccination in AIRD patients, differentiated by immunosuppressive medication. Composition of combination therapy groups is given in supplemental table 1. Dotted line marks the cut-off value for positivity following manufacturer's protocol (> 1 S/CO). CYC, Cyclophosphamide; GC, glucocorticoid; HCQ, hydroxychloroquine; JAKi, Janus kinase inhibitor; IL, interleukin; MMF, mycophenolate; mono, monotherapy; MTX, methotrexate; RTX ≤ 6 months, rituximab given ≤ 6 months prior to vaccination, RTX > 6 months, rituximab given > 6 months prior to vaccination; TNF, tumour necrosis factor.

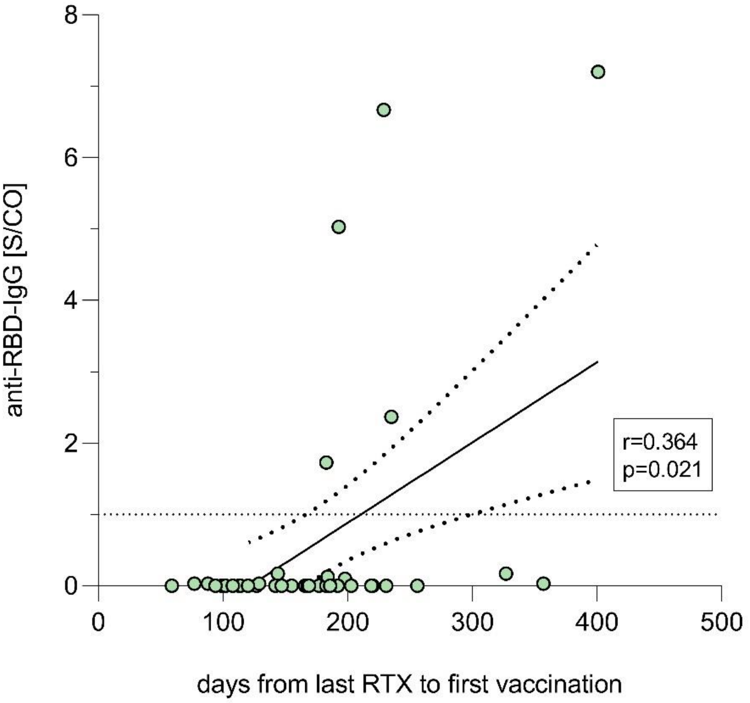

Supplemental figure 7: AIRD patients under rituximab (RTX) therapy (n=40). R and p according to Spearman's rank correlation between days from last RTX infusion to first vaccination and anti-RBD-IgG levels after second COVID-19 vaccination. Dotted line marks the cut-off value for positivity following manufacturer's protocol (> 1 S/CO).
